# Future health spending forecast in leading emerging BRICS markets in 2030: health policy implications

**DOI:** 10.1186/s12961-022-00822-5

**Published:** 2022-02-19

**Authors:** Mihajlo Jakovljevic, Demetrios Lamnisos, Ronny Westerman, Vijay Kumar Chattu, Arcadio Cerda

**Affiliations:** 1 Institute of Advanced Manufacturing Technologies, Peter the Great St. Petersburg Polytechnic University, St Petersburg, Russia; 2 Institute of Comparative Economic Studies, Hosei University, Tokyo, Japan; 3 Department of Global Health Economics and Policy, Faculty of Medical Sciences, University of Kragujevac, Kragujevac, Serbia; 4 Department of Health Sciences, European University Cyprus, Nicosia, Cyprus; 5 Federal Institute for Population Research, Wiesbaden, Germany; 6 Department of Medicine, Faculty of Medicine, University of Toronto, Toronto, ON M5G 2C4 Canada; 7 Center for Transdisciplinary Research, Saveetha Institute of Medical And Technical Sciences, Saveetha University, Chennai, India; 8 Department of Community Medicine, Faculty of Medicine, Datta Meghe Institute of Medical Sciences, Wardha, India; 9 Faculty of Economics and Business, University of Talca, Talca, Chile

**Keywords:** BRICS, Forecast, 2030, Sustainable Development Goals, UN, Brazil, Russia, India, China, South Africa, Health expenditure, Projections, Spending, Health policy, Health financing

## Abstract

**Background:**

The leading emerging markets of Brazil, Russia, India, China and South Africa (BRICS) are increasingly shaping the landscape of the global health sector demand and supply for medical goods and services. BRICS’ share of global health spending and future projections will play a prominent role during the 2020s. The purpose of the current research was to examine the decades-long underlying historical trends in BRICS countries’ health spending and explore these data as the grounds for reliable forecasting of their health expenditures up to 2030.

**Methods:**

BRICS’ health spending data spanning 1995–2017 were extracted from the Institute for Health Metrics and Evaluation (IHME) Financing Global Health 2019 database. Total health expenditure, government, prepaid private and out-of-pocket spending per capita and gross domestic product (GDP) share of total health spending were forecasted for 2018–2030. Autoregressive integrated moving average (ARIMA) models were used to obtain future projections based on time series analysis.

**Results:**

Per capita health spending in 2030 is projected to be as follows: Brazil, $1767 (95% prediction interval [PI] 1615, 1977); Russia, $1933 (95% PI 1549, 2317); India, $468 (95% PI 400.4, 535); China, $1707 (95% PI 1079, 2334); South Africa, $1379 (95% PI 755, 2004). Health spending as a percentage of GDP in 2030 is projected as follows: Brazil, 8.4% (95% PI 7.5, 9.4); Russia, 5.2% (95% PI 4.5, 5.9); India, 3.5% (95% PI 2.9%, 4.1%); China, 5.9% (95% PI 4.9, 7.0); South Africa, 10.4% (95% PI 5.5, 15.3).

**Conclusions:**

All BRICS countries show a long-term trend towards increasing their per capita spending in terms of purchasing power parity (PPP). India and Russia are highly likely to maintain stable total health spending as a percentage of GDP until 2030. China, as a major driver of global economic growth, will be able to significantly expand its investment in the health sector across an array of indicators. Brazil is the only large nation whose health expenditure as a percentage of GDP is about to contract substantially during the third decade of the twenty-first century. The steepest curve of increased per capita spending until 2030 seems to be attributable to India, while Russia should achieve the highest values in absolute terms. Health policy implications of long-term trends in health spending indicate the need for health technology assessment dissemination among the BRICS ministries of health and national health insurance funds. Matters of cost-effective allocation of limited resources will remain a core challenge in 2030 as well.

## Background

Most of the past three centuries of the world’s economic history, including the peak of the colonial era and the dawn of the first Industrial Revolution, has been characterized by a distinct pattern in the global economic hierarchy. Initial sparks of four consecutive industrial revolutions [1] originated primarily within European states and their colonial descendent cultures [2]. Typically, industrialized countries of the North invested their knowledge, financial resources and technology to establish manufacturing chains within the countries of the Global South [3]. There, high-quality goods and services, reliant on a local skilled, decently educated and relatively affordable local labour force, were usually consumed or exported back to the rich North [4].

Yet profound evolutionary changes in the world marketplace affecting the relationship between the rich industrialized Northern Hemisphere and underdeveloped Global South began to take place deep into the Cold War era, during the early 1980s [5]. Many of these changes unfolded far more rapidly after 1991 well into the era of accelerated globalization [6]. Ultimately, they began to reach maturity during the first two decades of the twenty-first century, becoming apparent to both the passionate protagonists of economic globalization [7] and its fierce opponents among leading economic and geopolitical voices [8]. The broadly accepted stratification of nations over almost half a century of the Cold War was one that split countries into the three distinctive layers, given the maturity of their overall development at the time. The First World referred to the leading Organisation for Economic Co-operation and Development (OECD) nations mostly situated within the Collective or Political West [9]. They were all free-market economies and managed to establish prosperous welfare societies generally up to the 1960s, including Japan [10]. The Asian Tiger economies, Greece, Finland, Israel and a few minor exceptions managed to achieve the same during the 1980s [11]. The Second World referred to the Union of Soviet Socialist Republics (USSR) and Warsaw Pact nations, whose economies were based on centrally planned communism [12]. Some of these nations, notably the USSR, also managed to achieve industrialization on a massive scale based on effective five-year plans [13]. This same long-term planning strategy was later adopted by the People’s Republic of China [14], and decades later remained at the core of the Chinese economic miracle [15]. Soviet technology lagged slightly behind the Western level, but in some areas such as aerospace technology [16] it was at the cutting edge of global innovation [17]. The Third World comprised most of the other low- and middle-income countries (LMICs) scattered across Africa, Latin America and Asia [18].

It was among these Third World LMICs, caught between the superpower rivalry, that the rise of the Non-Aligned Movement occurred [19]. Although neglected today, it once gave birth to the group of countries that would later be known as newly industrialized economies [20]. Fewer than 20 of these countries, whose pace of real gross domestic product (GDP) growth significantly outpaced that of mature high-income countries, were recognized as emerging markets [21]. This diverse and heterogeneous group of countries inherited an array of historical legacies in healthcare establishment, provision and financing [22]. The most important and largest among them, with significant global outreach, became known as the BRICS (Brazil, Russia, India, China, South Africa) [23]. These five countries represent around 25% of the world’s gross national income, more than 40% of the world’s population and about 40% of the global burden of disease (GBD) (Acharya et al.) Their aggregated GDP over the global GDP (in 2011 international dollars) increased from 17.6% in 1995 to 32.5% in 2018. It is projected that by 2050, China and India will become the first and third largest economies in the world, while Russia and Brazil will rank fifth and sixth, respectively, behind Japan (Pieterse 2012; Siddiqui 2016). They are also well positioned to exert a significant influence on global health, and their priorities on healthcare and financing are different from those of the OECD countries (Acharya et al. 2013). The purpose of this study is to closely explore long-term trends in fiscal flows intended for healthcare based on the existing GBD and Institute for Health Metrics and Evaluation (IHME)’s legacy and historical assessments. The purpose of such research effort is to make highly reliable forecasts of health expenditure patterns among the BRICS up to 2030. This particular time horizon was chosen to extend future projections as much as possible and preserve the substantial probability of scenarios, given the methodological framework and best data available. Also, 2030 is a convenient cross section given the fact that United Nations (UN)-endorsed Sustainable Development Goals (SDGs) [24] 3 and 10, namely good health and well-being (related to healthcare) and reduced inequality, as well as Agenda 2030 and its commitments related to public health, foresee precise tracking of individual countries’ achievements over this time frame [25]. This study will also offer significant and novel insights and will bridge gaps in the seminal literature as the best BRICS health expenditure forecasts up to 2025 available to date [26]. More profound research questions underlying this effort relate to the assessment of their impact on the global demand and supply of healthcare-related goods and services in a post-COVID-19 world market [27].

This work will analyse the health policy implications of current challenges to increasing health spending in order to meet growing citizen demand for medical services and pharmaceuticals across the BRICS countries. Our goal is to better understand the current trends and reveal the hidden patterns of expenditure and financing in these rapidly growing economies. Lastly, but importantly, we will attempt to propose a few effective strategies for coping with continuing resource constraints and need for cost containment. Such issues remain high on national policy-makers’ agenda, and even much higher in comparison with traditionally rich high-income economies. Even the percentage of the national budgetary allocation for healthcare in BRICS remains significantly lower relative to leading Western and Asian OECD sectors. Combined with the growing burden of noncommunicable diseases (NCDs; roughly 75% of global NCD burden in terms of disability-adjusted life-years is attributable to the LMICs) and less effective funding mechanisms, the size and scope of this challenge is clearly huge. Beyond their economic relevance, BRICS have also gained demographic importance, as these five nations account for more than 40% of the world’s population, and about 20% of the world’s urban middle-class population (do Nasciemento 2020).

## Methods

### Data sources and data

We extracted health spending data for the BRICs countries for the period spanning 1995 to 2017 from the IHME’s Financing Global Health 2019 database [28–31]. These data track government health spending from domestic sources, including general budget support and social health insurance; prepaid private health spending, which includes private insurance and nongovernmental organization spending; out-of-pocket health spending, which consists of all expenditures at the point of service including copayments; and developmental assistance for health. The sum of these sources makes up total healthcare spending. All health spending and all-sector government spending estimates from this database are reported in inflation-adjusted 2019 purchasing power parity (PPP)-adjusted US dollars.

### Statistical analysis

Mean and standard deviation were used to summarize past health spending and government, prepaid private and out-of-pocket spending from 1995 to 2017. We estimated future total health spending and government, prepaid private and out-of-pocket health spending per capita and as a percentage of GDP from 2018 to 2030. We also estimated government, prepaid private and out-of-pocket spending as a share of the total health spending from 2018 to 2030. The methods used for this projection are based on time series analysis and autoregressive integrated moving average [ARIMA](p,d,q) models, where (p,d,q) are the autoregressive order, the degree of differencing and the moving average order [32–34]. ARIMA models use retrospective health spending data from 1995 to 2017 to forecast future values and trends. These models are the most general class for forecasting a time series which can be made to be stationary by differencing in conjunction with nonlinear transformations such as logging or deflating.

The best ARIMA model was selected for each health spending source and each country using the auto.arima function in R [34]. This function uses a variation of the Hyndman–Khandakar algorithm [35], which combines unit root tests, minimization of the Akaike information criterion (AIC) and maximum likelihood estimation (MLE) to obtain the best ARIMA model. The unit root test is a test of stationarity and it is used to determine whether differencing is required in the ARIMA models. The null hypothesis is that the data are stationary, and a statistically significant result suggests that differencing is necessary in the model. The AIC is an estimator of prediction error and therefore assesses the relative quality of statistical models for a given set of data. Given a set of possible models for fitting the data, the AIC estimates the quality of each model relative to each of the other models and therefore provides a means for model selection. The analysis was performed with R (version 3.3.1), and 95% confidence levels for prediction intervals are reported. The results of the best-fitting ARIMA models for each health spending and country scenario are presented in Appendix.

## Results

Table 1 presents the mean and standard deviation for health spending per capita and as a proportion of GDP, and the source of health spending per capita and share of health spending for the years 1995 to 2017. The highest value of health spending per GDP in the years 1995 to 2017 was observed for Brazil (8.4%), with the highest share of spending attributed to government spending (42.7%). The countries with the next highest health spending per GDP during 1995–2017 were South Africa (7.5%) and the Russian Federation (5.1%), both countries having government spending as the main source of health spending (45.4% and 61.6% of the total health spending, respectively). China and India had the lowest health spending per GDP during 1995–2017 (4.2% for China and 3.7% for India), both having out-of-pocket spending as the main source of health spending (China 49.1% and India 69.2%).

**Table 1:** Descriptive statistics (mean and standard deviation) for health spending per capita and as a proportion of GDP, source of health spending per capita and share of health spending for the years 1995 to 2017

	Health spending per capita ($)	Health spending per GDP (%)	Source of health spending			Share of health spending		
			Government spending per capita ($)	Prepaid private spending per capita ($)	Out-of-pocket spending per capita ($)	Government spending as share of total (%)	Prepaid private spending as share of total (%)	Out-of-pocket spending as share of total (%)
Brazil	1236.0 (158.3)	8.2 (0.4)	529.0 (77.1)	298.7 (81.8)	406.2 (6.2)	42.7 (1.0)	23.7 (3.4)	33.4 (4.1)
China	373.0 (239.7)	4.2 (0.4)	178.3 (160.3)	30.7 (8.5)	163.7 (73.3)	39.9 (13.5)	10.8 (5.0)	49.1 (9.5)
India	159.1 (48.4)	3.7 (2.02)	36.3 (15.7)	11.9 (6.1)	108.6 (26.6)	22.2 (3.0)	7.0 (1.4)	69.2 (4.1)
Russian Federation	1152.4 (281.7)	5.1 (0.4)	707.0 (164.0)	60.2 (16.2)	384.4 (131.7)	61.6 (2.8)	5.6 (2.3)	32.6 (3.7)
South Africa	934.5 (155.1)	7.5 (0.6)	434.6 (139.4)	377.3 (28.7)	106.3 (16.8)	45.4 (7.4)	41 (5.3)	11.9 (3.5)

**Fig. 1:**
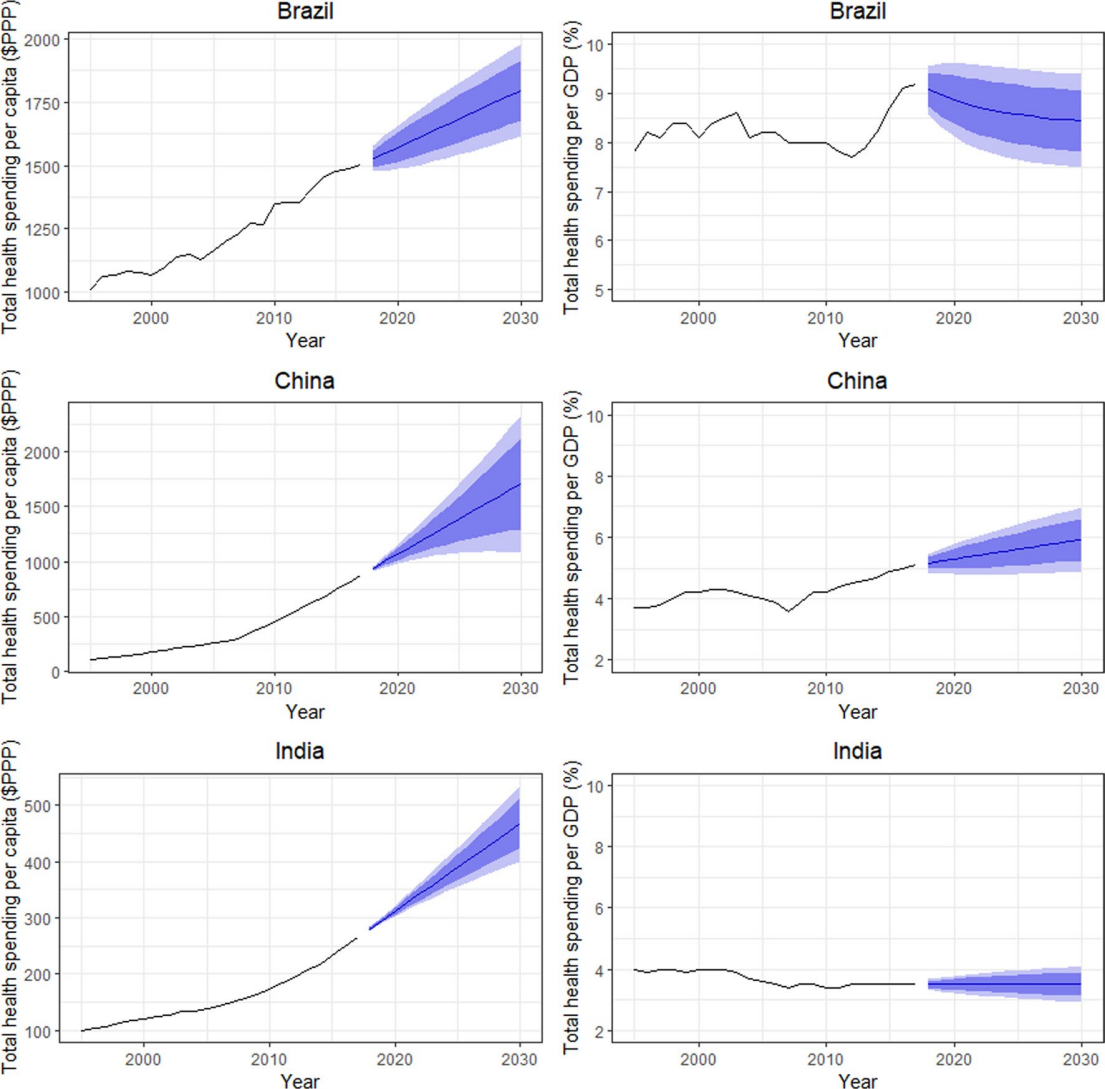
Total health spending per capita in inflation-adjusted 2019 PPP-adjusted US dollars and total health spending as a percentage of GDP for Brazil, China and India

**Fig. 2:**
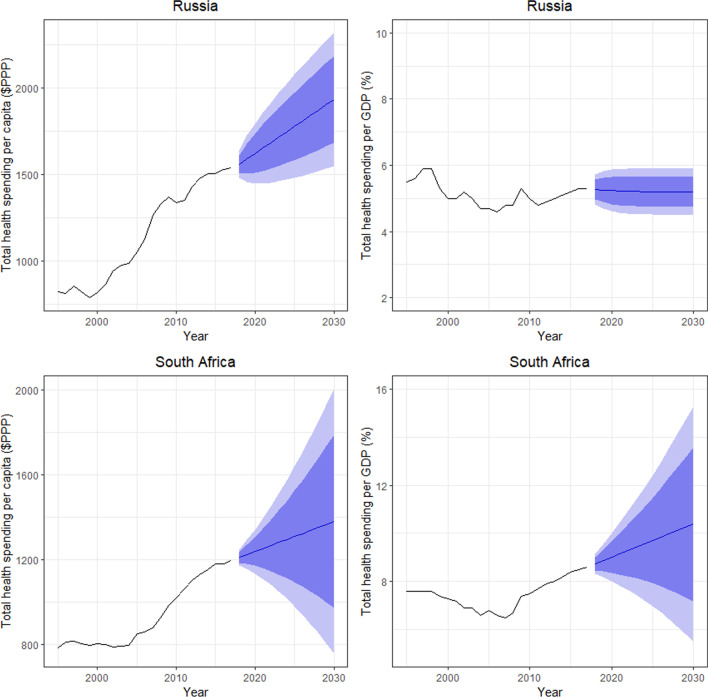
Total health spending per capita in inflation-adjusted 2019 PPP-adjusted US dollars and total health spending as a percentage of GDP for Russia and South Africa

Figures 1 and 2 show the total health spending per capita in inflation-adjusted 2019 PPP-adjusted US dollars and total health spending as a share of GDP for the BRICS countries. These figures show how per capita health spending is expected to increase between 2017 and 2030. This growth is adjusted for inflation and PPP. Health spending is projected to be highest in Brazil, China and Russia, which already spend the most on health. Health spending in 2030 is projected to be $1767 (95% prediction interval [PI] 1615, 1977) for Brazil, $1707 (95% PI 1079, 2334) for China, $1933 (95% PI 1549, 2317) for Russia, $1379 (95% PI 755, 2004) for South Africa and $468 (95% PI 400.4, 535) for India (Table 1). Health spending per GDP in 2030 is projected to be highest in Brazil and South Africa, followed by China and Russia, and lastly India. Health spending per GDP is projected to increase in South Africa and be 10.4% by 2030 (95% PI 5.5, 15.3), while health spending per GDP is projected to decrease slightly in Brazil to 8.4% in 2030 (95% PI 7.5, 9.4) (Table 2). China is also expected to see a steady increase in health spending per GDP from 2020 to 2030, and it is projected to be 5.9% (95% PI 4.9, 7.0) in 2030. Russia and India are not expected to experience any noticeable change in their health spending per GDP during the time horizon up to 2030, and health spending per GDP in 2030 is projected to be 5.2% (95% PI 4.5, 5.9) for Russia and 3.5% (95% PI 2.9%,4.1%) for India.

**Table 2:** Expected health spending per capita and as a proportion of GDP for the years 2020, 2025 and 2030

	2020		2025		2030	
	Health spending per capita ($)	Health spending per GDP (%)	Health spending per capita ($)	Health spending per GDP (%)	Health spending per capita ($)	Health spending per GDP (%)
Brazil	1572 (1485, 1659)	8.9 (8.1, 9.6)	1684 (1542, 1826)	8.6 (7.7, 9.5)	1796 (1615, 1977)	8.4 (7.5, 9.4)
China	1067 (985, 1149)	5.3 (4.8, 5.8)	1387 (1074, 1700)	5.6 (4.8, 6.4)	1707 (1079, 2334)	5.9 (4.9, 7.0)
India	311 (301, 321)	3.5 (3.2, 3.8)	390 (355, 424)	3.5 (3.0, 4.0)	468 (400.4, 535)	3.5 (2.9, 4.1)
Russian Federation	1621 (1449, 1794)	5.2 (4.6, 5.9)	1777 (1480, 2075)	5.2 (4.5, 5.9)	1933 (1549, 2317)	5.2 (4.5, 5.9)
South Africa	1237 (1134, 1340)	9.0 (8.0, 10.0)	1308 (980, 1637)	9.7 (7.0, 12.4)	1379 (755, 2004)	10.4 (5.5, 15.3

The sources of health spending per capita and their respective share of health spending for the years 2020, 2025 and 2030 are displayed in Tables 3 and 4, respectively. The highest government health spending per capita in 2020 is observed for Russia, at $871 (95% PI 716, 1028), followed by Brazil at $663 (95% PI 616, 711), South Africa at $648 (95% PI 578, 719), China at $609 (95% PI 553, 664) and lastly India at $85 (95% PI 76, 94). In 2030, it is expected that the highest government health spending per capita will be observed for China, at $949 (95% PI 527, 1370), followed by Russia at $870 (95% PI 428, 1312), Brazil at $761 (95% PI 662, 860), South Africa at $665 (95% PI 311, 1020) and India at $135 (95% PI 69, 201) (Table 3). In terms of the share of government health spending, the highest value in 2020 is observed for Russia, with 58.2% (95% PI 55.2, 61.2), followed by China with 57.6% (95% PI 52.2, 63.0), South Africa with 52.9% (95% PI 48.1, 57.8), Brazil with 41.7% (95% PI 40.0, 43.2), and lastly India with 27.4% (95% PI 25.1, 29.7). None of the countries is expected to experience any noticeable change in government health spending share up to 2030. In 2030, the share of government health spending for Russia is projected to be 63.2% (95% PI 57.3, 69.2), followed by China with 57.0% (95% PI 25.6, 88.4), South Africa with 53.0% (95% PI 31.8, 74.2), Brazil with 41.6% (95% PI 36.9, 46.2) and India with 30.7 (95% PI 25.4, 36.0) (Table 4).

**Table 3:** Source of health spending per capita for the years 2020, 2025 and 2030

	2020			2025			2030		
	Government spending per capita ($)	Prepaid private spending per capita ($)	Out-of-pocket spending per capita ($)	Government spending per capita ($)	Prepaid private spending per capita ($)	Out-of-pocket spending per capita ($)	Government spending per capita ($)	Prepaid private spending per capita ($)	Out-of-pocket spending per capita ($)
Brazil	663 (616, 711)	504 (468, 539)	406 (394, 418)	713 (635, 790)	585 (496, 675)	406 (393, 418)	761 (662, 860)	667 (514, 820)	406 (393, 418)
China	609 (553, 664)	81 (71, 91)	370 (348, 393)	779 (569, 989)	121 (84, 158)	472 (395, 548)	949 (527, 1370)	161 (86, 235)	573 (425, 719)
India	85 (76, 94)	32 (29, 36)	190 (179, 200)	110 (77, 143)	42 (32, 53)	229 (193, 266)	135 (69, 201)	53 (34, 72)	268 (198, 339)
Russian Federation	871 (716, 1028)	40 (18, 63)	670 (625, 715)	870 (544, 1197)	40.4 (0.8, 80.1)	759 (672, 845)	870 (428, 1312)	40 (1, 92)	848 (733, 963)
South Africa	648 (578, 719)	444 (396, 492)	96 (85, 107)	660 (445, 875)	445 (350, 539)	101 (58, 144)	665 (311, 1020)	445 (318, 571)	106 (19, 193)

**Table 4:** Share of health spending for the years 2020, 2025 and 2030

	2020			2025			2030		
	Government spending as share of total (%)	Prepaid private spending as share of total (%)	Out-of-pocket spending as share of total (%)	Government spending as share of total (%)	Prepaid private spending as share of total (%)	Out-of-pocket spending as share of total (%)	Government spending as share of total (%)	Prepaid private spending as share of total (%)	Out-of-pocket spending as share of total (%)
Brazil	41.7 (40.0, 43.2)	32.4 (31.3, 33.5)	25.9 (23.7, 27.0)	41.6 (38.2, 45.0)	36.0 (32.6, 39.5)	23.3 (20.9, 25.6)	41.6 (36.9, 46.2)	39.7 (33.3, 46.1)	20.6 (17.7, 23.6)
China	57.6 (52.2, 63.0)	8.0 (6.2, 9.8)	34.3 (28.5, 40.1)	57.2 (38.8, 75.6	10.5 (3.5, 17.5)	32.4 (12.9, 52.6)	57.0 (25.6, 88.4)	13.0 (1.0, 27.1)	30.4 (3.0, 79.9)
India	27.4 (25.1, 29.7)	10.9 (10.1, 11.6)	60.9 (58.2, 63.7)	29.0 (24.9, 33.1)	12.5 (10.4, 14.5)	58.3 (53.4, 63.2)	30.7 (25.4, 36.0)	14.0 (10.4, 17.7)	55.7 (49.3, 62.0)
Russian Federation	58.2 (55.2, 61.2)	2.6 (0.5, 4.7)	42.3 (39.5, 45.1)	64.2 (59.0, 69.5)	2.6 (0.3, 8.2)	45.7 (40.1, 51.3)	63.2 (57.3, 69.2)	2.6 (0.0, 11.1)	49.0 (41.6, 56.4)
South Africa	52.9 (48.1, 57.8)	36.8 (32.0, 41.5)	7.8 (6.4, 9.2)	53.0 (38.1, 67.8)	36.7 (23.5, 49.9)	7.8 (2.6, 13.0)	53.0 (31.8, 74.2)	36.7 (18.9, 54.5)	7.8 (1.0, 18.3)

In terms of prepaid private spending per capita, the highest spending in 2020 is expected to be observed by Brazil, at $504 (95% PI 468, 539), followed by South Africa at $444 (95% PI 396, 492), China at $82 (95% PI 71, 91), Russia at $40 (95% PI 18, 63) and India at $32 (95% PI 29, 36). In 2030, the prepaid private spending per capita in Brazil is expected to increase and to again be the highest, at $667 (95% PI 514, 820), followed by South Africa at $445 (95% PI 318, 571), China at $161 (95% PI 86, 235), India at $53 (95% PI 34, 72) and lastly Russia at $40 (95% PI 1, 92) (Table 3). In terms of the share of prepaid private health spending, the highest in 2020 is expected to be observed by South Africa, with 36.8% (95% PI 32.0, 41.5), followed by Brazil with 32.4% (95% PI 31.3, 33.5), India with 10.9% (95% PI 10.1, 11.5), China with 8.0% (95% PI 6.2, 9.8) and Russia with 2.6% (95% PI 0.5, 4.7). In 2030, Brazil and South Africa are expected to have the highest share of prepaid private spending, with 39.7% (95% PI 33.3, 46.1) for Brazil and 36.7% (95% PI 18.9, 54.5) for South Africa. China and India are expected to have a much smaller share of prepaid private spending, 14.0% (95% PI 10.4, 17.7) for India and 13.0% (95% PI 1.0, 27.1) for China, while Russia is expected to have the lowest share of prepaid private spending, at 2.6% (95% PI 0.0, 11.1) (Table 4).

The highest out-of-pocket spending per capita in 2020 is expected to be observed by Russia, at $670 (95% PI 625, 715), followed by Brazil at $406 (95% PI 394, 418), China at $370 (95% PI 348, 393), India at $190 (95% PI 179, 200) and lastly South Africa at $96 (95% PI 85, 107). In 2030, the out-of-pocket spending per capita in Russia is expected to increase and to again be the highest, at $848 (95% PI 733, 763), followed by China at $573 (95% PI 425, 719), Brazil at $406 (95% PI 393, 418), India at $268 (95% PI 198, 339) and South Africa at $106 (95% PI 19, 193) (Table 3). In terms of the out-of-pocket share of health spending, the highest in 2020 is expected to be observed by India at 60.9% (95% PI 58.2, 63.7), followed by Russia at 42.3% (95% PI 39.5, 45.1), China at 34.3% (95% PI 28.5, 40.1), Brazil at 25.9% (95% PI 23.7, 27.0) and South Africa at 7.8% (95% PI 6.4, 9.2). In 2030, India and Russia are expected to have the highest out-of-pocket spending share, 55.7% (95% PI 49.3, 62.0) for India and 49.0% (95% PI 41.6, 56.4) for Russia, followed by China with 30.4% (95% PI 3.0, 79.9), Brazil with 20.6% (95% PI 17.7, 23.6) and South Africa with 7.8% (95% PI 1.0, 18.3) (Table 4).

### Strengths and limitations

We have used ARIMA models to estimate total health, government, prepaid private and out-of-pocket spending per capita and as a share of GDP from 2018 to 2030, as well as the share of health spending by source. ARIMA models are the most general class for forecasting a time series, and we have used the best-fitting ARIMA model for the data for each country and health spending source using an automatic process [34, 35]. These models are a general class of time series models and provide useful forecasts of future time series; however, they have some limitations in the case of forecasting health spending. First, there are some cases where we observed high uncertainty in forecasting values, as indicated by the large prediction interval, and this may be attributed to the sparse available retrospective data for each country and the uncertainty in specifying the pattern and trend of the past values. The available data for each country and each source of health expenditure were from the period 1995 to 2017. For some cases, there was not an obvious pattern in the data to help predict future values with low uncertainty (i.e. small prediction intervals of future values).

Moreover, the underlying retrospective data include some measurement error and imputation [30]. Precise data that are comparable and complete across a long period and for all countries are not available. Finally, any estimation of future health spending is vulnerable to national and international policy decision-making, the supply and demand of the health system, economic development, natural disasters, pandemics, war and other environmental issues potentially related to climate change. Because the forecasting of health spending, in general, is far from exact, we quantify uncertainty with prediction intervals that increased the further we projected into the future.

## Discussion

The five emerging economies observed in this work exhibited distinctively different underlying patterns leading to the observed historical health spending [36]. Each of these countries had experienced their unique turning point in recent history. While we are able to track many decades of historical business cycles [37] of real GDP expansion and contraction for most countries worldwide, the accessibility of health expenditure data is much different. Accounting practices are diverse and country-specific, which leads to serious issues compromising the comparability of older data [38]. Japan, for instance, accounts for social support and long-term care for elderly citizens outside official health spending statistics (almost 10% of entire consumption), while Canada does exactly the opposite. Even within OECD countries, this leads to substantial distortions of comparability which make comparative research difficult and less reliable. For example, the share of long-term health spending ranges from 24.9% in Portugal to 92.5% in Spain among countries with highly similar populations and health system traditions [39]. Also, most of the former socialist countries of Eastern Europe and the USSR republics lack distinction of observed subsegments of health spending for lengthy periods before the mid-1990s [40]. The first broadly accepted attempt to track fiscal flows within the national health systems with great accuracy and satisfactory transnational comparability was WHO’s introduction of national health accounts (NHA) [41]. This system of tracking medical care-attributable spending dates back only to 1995. It was officially endorsed and ratified by almost 190 UN member countries. It was further fine-tuned and stratified across subsections of expenditures, and this updated version was embraced by the Global Health Expenditure Database (GHED), whose official release so far covers the period from 2000 to 2018 [42]. Thus, we can discuss and draw reliable conclusions on historical trends in health spending preceding our 2020–2030 forecast, mostly for the period 1995–2020, with a satisfactory extent of transitional comparability among inherently different economic and health systems.

Brazil has experienced several business cycles since the early 1990s that have driven its economic growth upward and downward, with several shifts. It entered the BRIC counties in 1995, being the wealthiest country in the group by far, exceeding others in terms of per capita health expenditures in both nominal and PPP terms. Over time, it has changed considerably, and was surpassed by Russia during the early 2000s. Unlike most other members of the group, it is characterized by a more robust private insurance sector among its upper-income layers of society [43]. To maintain that economic stability, annual GDP growth of 3.3% is needed for Brazil (Pendraza et al. 2018). More challenging will be reforms in social security and economic growth to face the ageing of the population; otherwise, fewer young Brazil workers will need to compensate for the fiscal gap.

Russia’s legal predecessor in international law, the USSR, was the second-ranked global economy behind the United States for most of the 1950s–1970s. After the dissolution of the USSR in 1991, Russia was driven into one of the world’s most severe economic recessions of the twentieth century [44], dragging down the majority of formerly mutually dependent centrally planned Eastern European and Central Asian economies, reaching its bottom in 1998. Therefore, its public and governmental health spending in the middle of the 1990s was exceptionally low, while out-of-pocket spending grew tremendously [45]. After painful consolidation of the free-market capitalism in the country, the situation has rapidly improved in the early 2000s. The share of governmental responsibility for health spending and support for the unemployed, children and the elderly have become much more generous, decreasing the vulnerability of rural and lower-income citizens to catastrophic health spending [46]. The long-term dynamics of the Russian economy have revealed a large margin of resistance to the volatilities [47] in the global market, including the COVID-19-induced recession that has just begun. Thus, the broadly accepted academic consensus and assessment of most domestic and foreign analysts is that the country will be able to achieve universal health coverage milestones for most of its population [48]. Within in the BRICS group, Russia has to struggle the most with the demographic impact of progressive ageing and lower fertility rates compared to other BRICS countries (United Nations Department of Economic and Social Affairs, 2019).

India’s health sector is quite a unique representative of the group, with several distinctive features. India has had a free market economy since 1991. It is the only country among the four large BRIC counties whose demographic dividend has not yet been consumed. India is at a far younger stage of its population ageing, and thus suffers from a much lower burden of NCDs [49]. Given its fertility levels, it is going to experience an expansion of its labour force by up to 150 million young and well-educated people [50]. Its health system has long been marked by substantial heterogeneity among its federal states and lack of strong federal control [51]. This fiscal control has become far more stringent in recent years [52]. The respective younger population is characterized by a lower share of elderly citizens and better old-age dependency ratios. Alongside India’s strong real economic growth rates and traditional family caregiving [53], these facts shape India’s demand for medical care and the structure of spending. However, the affordability issues of access to hospital care and essential medicines remain high for its vast rural populations. To make the landscape more complex, India’s generic drug manufacturing industry is a globally competitive one. Ranbaxy and other giant Indian companies have achieved and maintained profound market penetration of their products in almost 200 countries worldwide, including the most strongly regulated high-income Asian markets with exceptionally high quality thresholds [54]. On the other hand, India needs to respond to specific challenges in overpopulation and internal migration dynamics. There is an imbalance in population growth due to locally concentrated urbanization in provinces such Chandigarh and Delhi, where more than 80% of all urbanized people live. In contrast, provinces like Bihar and Sikkim have a much lower economic concentration and minor urbanized population (do Nascimento 2020).

China’s most well-known historical turning point since the establishment of the People’s Republic back in 1949 was the introduction of profound social reforms under Deng Xiaoping around 1978 [55]. As these reforms matured, we can track almost continuous strong upward Chinese economic growth since 1989 [56]. A short-lived slowdown in real GDP growth took place during the COVID-19-induced lockdown and massive quarantines of large cities and intraregional travel. Nevertheless, this changed rapidly after the pandemic was effectively managed in this huge nation [57]. China continues to be the major driver of global and Asian economic growth, culminating with the recent establishment of the world’s largest free trade zone, the Regional Comprehensive Economic Partnership, or RCEP [58]. Several factors have driven significant setbacks for the huge Chinese health system. The decades-old one-child policies have distorted the national demographic pyramid, creating conditions for accelerated population ageing [59].

As we approach 2050, China will exceed Japan, becoming the fastest-ageing large nation worldwide [60]. The hospital sector in many rich coastal industrial provinces essentially funds itself through a large margin between wholesale and retail prices of medicines as a result of massive public procurement procedures [61]. The domestic pharmaceutical manufacturing sector is powerfully developed yet mostly focused on traditional domestic Chinese medicinal products. Its ability to patent and sell brand-name innovative pharmaceuticals beyond domestic and regional markets remains somewhat limited [62]. Yet the scale of the Chinese pharmaceutical market is enormous. Recently it has surpassed the Japanese market, which was second-ranked per value-based turnover globally for decades. All other sectors of Chinese healthcare are following its continued rapid urbanization and industrialization. Demand and domestic manufacturing of imaging diagnostics and other medical devices is growing at a double-digit pace [63]. The compound annual growth rates (CAGR) of the Chinese domestic market in terms of uptake of foreign-patented brand-name pharmaceuticals and vaccines continue to be far higher than those of any mature high-income OECD countries [64]. However, with most of the Industry 4.0 e-Health applications, artificial intelligence developments and associated software algorithms and advanced 5G mobile network, it is the opposite scenario [65]. In this arena, huge Chinese global companies are positioned at the cutting edge of innovation [66] and even hold the majority of patents in comparison to other significant players [67]. Their domestic technology evolution is virtually driving the pace of innovation worldwide in some of these areas [68]. Given the large-scale applications in intensive medical care, and provision of services to the elderly, vulnerable and citizens living in remote areas, these are likely to be drivers of the enormous growth in domestic demand for healthcare goods and services. The current dynamics, in light of the increasing trade and intellectual property disputes between China and the United States, nicknamed the “Cold War 2.0” [69], are likely to shape revenue streams on exports of such technologies worldwide [70]. The overall impression supported by our forecasts is one of the growing ability of this country to increase its long-term investment in healthcare. Such a trend appears to be sustainable in both national and per capita terms in the end regardless of several possible scenarios of the struggle for achieving multipolarity and fostering Belt and Road policies [71].

As the final letter in the BRICS acronym, South Africa was the last to officially join the group in 2008 as part of a multilateral agreement on trade and cooperation [72]. Since then, BRICS heads of state, ministers of health, chambers of commerce and business sectors continue to cooperate across increasingly broad areas of the economy. The South African member is not comparable to the others in terms of the size of the population or economy, but it has crucial complementarities in terms of resource exports. Also, it brings an important perspective as one of the engines of pan-African development during the twenty-first century. The famous historical turning point in the South African republic was Nelson Mandela’s victory in overthrowing the country’s Apartheid regime [73]. This paved the way for many future reforms. Based on our estimates, South Africa has the largest margins of uncertainty in the future given its historical peculiarities and specifics of demography, morbidity and mortality patterns in the African nations [74]. Obviously, among the BRICS it hosts by far the youngest population in the earliest stage of population ageing. Its health spending will be driven by an entirely different spectrum of needs, ranging from combating infectious disease to extensive development of health facility networks and increasing access to medical care for the broad layers of society. The major public health problems in South Africa are related to sexually transmitted diseases like HIV (20% of the adult population was infected with HIV in 2017) and a high maternal mortality rate—119 maternal deaths per 100,000 live births (WHO 2019). The population of South Africa will continue to grow for the next decades, with annual growth rates below 1%. The demographic dividend in South Africa will remain positive, with younger working people compared to other BRICS countries like Brazil or Russia.

## Health policy implications

Among the five BRICS countries, a few distinctively different pathways of health spending are visible. India and Russia are highly likely to remain stable in terms of total health spending share of GDP until 2030. In reality, this trend has been present in both nations with minor fluctuations over a decades-long time horizon. These forecasted values expose a significant degree of probability under an array of geopolitical scenarios [75]. In contrast, China, as the major driver of global economic growth associated with the broader ASEAN (Association of Southeast Asian Nations) region, will be capable of significantly expanding its investment in the health sector across an array of indicators [76]. The likelihood of its success in achieving targeted SDGs in terms of universal coverage are probably the highest among the group. All five BRICS demonstrate long-term trends of increasing per capita spending in PPP terms. Brazil, however, is probably the greatest surprise [77]. It appears it is the only large nation whose health expenditure as a share of GDP is poised to contract substantially during the third decade of the twenty-first century. The steepest increase in per capita spending until 2030 seems to be attributable to India, while Russia should achieve the highest values in absolute terms. An overall impression among the BRICS is that most of them are about to evolve out of their historical legacies in healthcare in a rather predictable manner. We believe these projections of health expenditures by some of the most influential global healthcare markets should contribute to informing policy-makers’ decisions on resource allocation. It should also provide a hint for further health economics research on BRICS health sectors.

Although their historical legacies in establishing health systems are very diverse, BRICS nations share several common challenges. The first is the rapidly growing burden of NCDs, with approximately 75% remaining within the world’s LMICs. This workload and its associated costs of prevention, diagnostics and care exists alongside an unliquidated pool of infectious communicable diseases. The second core societal transformation is the accelerated population ageing, giving rise to a shrinking base of taxpayers who contribute to the health insurance funds. The third challenge is the rapidly increasing penetration of cutting-edge medical technologies such as monoclonal antibodies, with budget impacts far exceeding the line of public affordability. To address these major bottleneck inefficiencies, far more effective policy strategies need to be developed. The goal of such programmes would be to narrow the widening gap between the rich and poor citizens and to secure equity in healthcare access to the greatest extent possible. Probably the core political debate within BRICS ministries of health and insurance funds is centred on achieving far more cost-effective resource allocation. To some extent, capacity-building of national health technology assessment policies might be the response. Yet such programmes have shown limited applicability in traditional health systems far outside the Western legacy. There are inner governing mechanisms and strategies to cope with these challenges, which might not appear to be so effective but are still able to resolve an array of issues ranging from drug shortages to the waiting times for expensive transplant surgery or home-based medical care for the elderly. These decision-making pathways also largely rely on informal or family caregiving and social solidarity, which brings to market workforce and capacities otherwise unaffordable to the public sector. Within most of the BRICS countries, prosperous urban, coastal and industrial populations of megacities and increased living standards have brought about a significant share of prosperity. This is also reflected in substantially increased longevity in China, Russia and elsewhere. A smooth demographic transition ahead for India will remain far milder given the fact that it is still in a rather juvenile stage of population ageing. It is still expected to harvest its demographic dividend, bringing up to 150 million of a young skilled labour force to the market as we approach the middle of the twenty-first century. However, in all of these countries, including India, a huge rural population remains that lacks access to technologically advanced hospital care and expensive branded pharmaceuticals. Most of the BRICS governments have adopted generic replacement policies to tackle “the drug bill” which, in contrast to the 15–25% share in the Western OECD, may account for as much as 50% of total health spending in vast regions of China. Most BRICS governments have now adopted an array of more or less ambitious reform programmes designed to improve cost-effective decision-making pathways, particularly for the approval of innovative medicines. This same process took up to four decades in prominent Asian economies such as Japan or South Korea. Whether the pace of legislative framework reforms will be sufficient to achieve the BRICS’ development agenda and SDGs of Agenda 2030 remains to be seen in the years to come.

Although the BRICS nations are formed as a cooperative group with emerging economies, each country has to face its specific challenges in maternal mortality, HIV and sexually transmitted diseases, ageing populations and heterogeneous urbanization that could affect economic growth.

## Data Availability

Not applicable.

## Appendix

See Tables

**Table 5:** Parameter estimates with their standard errors of the ARIMA(p,d,q) models for the years 1995 to 2017 for Brazil

	**Health** **spending** **per capita ($)**	**Health** **spending** **per GDP (%)**	**Source of health spending**			**Share of health spending**		
			Government spending per capita ($)	Prepaid private spending per capita ($)	Out-of-pocket spending per capita ($)	Government spending as share of total (%)	Prepaid private spending as share of total (%)	Out-of-pocket spending as share of total (%)
**Model**	ARIMA(0,1,0)	ARIMA(1,0,0)	ARIMA(0,1,0)	ARIMA(0,2,1)	ARIMA(0,0,1)	ARIMA(1,1,0)	ARIMA(0,2,1)	ARIMA(0,1,)
**Estimates (SE)**	Drift = 22.4 (5.3)	AR1 = 0.85 (0.13) Mean = 8.3 (0.29)	Drift = 9.82 (2.92)	MA1 = −0.77 (0.12)	MA1 = 0.69 (0.17)	AR1 = 0.55 (0.18)	MA1 = −0.50 (0.22)	Drift = −0.53 (0.09

ARIMA(p,d,q): Autoregressive integrated moving average model and (p,d,q) are the autoregressive order, the degree of differencing and the moving average order

SE: standard error

AR: Autoregressive parameter

MA: Moving average parameter

5,

**Table 6:** Estimates with their standard errors of the ARIMA models for the years 1995 to 2017 for China

	**Health** **spending** **per capita ($)**	**Health** **spending** **per GDP (%)**	**Source of health spending**			**Share of health spending**		
			Government spending per capita ($)	Prepaid private spending per capita ($)	Out-of-pocket spending per capita ($)	Government spending as share of total (%)	Prepaid private spending as share of total (%)	Out-of-pocket spending as share of total (%)
**Model**	ARIMA(0,2,0)	ARIMA(0,1,0)	ARIMA(0,2,0)	ARIMA(0,2,0)	ARIMA(0,2,1)	ARIMA(1,1,1)	ARIMA(0,2,0)	ARIMA(0,2,1)
**Estimates (SE)**		Drift = 0.06 (0.03)			MA1 = −0.37(0.19)	AR1 = 0.88 (0.08) MA1 = 0.45(0.25)		MA1 = −0.49 (0.23)

ARIMA(p,d,q): Autoregressive integrated moving average model and (p,d,q) are the autoregressive order, the degree of differencing and the moving average order

SE: standard error

AR: Autoregressive parameter

MA: Moving average parameter

6,

**Table 7:** Estimates with their standard errors of the ARIMA models for the years 1995 to 2017 for India

	**Health** **spending** **per capita ($)**	**Health** **spending** **per GDP (%)**	**Source of health spending**			**Share of health spending**		
			Government spending per capita ($)	Prepaid private spending per capita ($)	Out-of-pocket spending per capita ($)	Government spending as share of total (%)	Prepaid private spending as share of total (%)	Out-of-pocket spending as share of total (%)
**Model**	ARIMA(1,2,0)	ARIMA(0,1,0)	ARIMA(0,2,0)	ARIMA(0,2,1)	ARIMA(0,2,1)	ARIMA(0,1,1)	ARIMA(0,2,1)	ARIMA(0,1,1)
**Estimates (SE)**	AR1 = −0.61 (0.16)			MA1 = −0.55 (0.17)	MA1 = −0.35 (0.19)	MA1 = 0.73 (0.17) Drift = 0.33 (0.15)	MA1 = −0.72 (0.15)	MA1 = 0.64 (0.15) Drift = −0.53 (0.18)

ARIMA(p,d,q): Autoregressive integrated moving average model and (p,d,q) are the autoregressive order, the degree of differencing and the moving average order

SE: standard error

AR: Autoregressive parameter

MA: Moving average parameter

7,

**Table 8:** Estimates with their standard errors of the ARIMA models for the years 1995 to 2017 for the Russia Federation

	**Health** **spending** **per capita ($)**	**Health** **spending** **per GDP (%)**	**Source of health spending**			**Share of health spending**		
			Government spending per capita ($)	Prepaid private spending per capita ($)	Out-of-pocket spending per capita ($)	Government spending as share of total (%)	Prepaid private spending as share of total (%)	Out-of-pocket spending as share of total (%)
**Model**	ARIMA(0,1,1)	ARIMA(1,0,0)	ARIMA(1,1,)	ARIMA(0,1,1)	ARIMA(1,1,0)	ARIMA(2,0,0)	ARIMA(1,1,0)	ARIMA(1,1,0)
**Estimates (SE)**	MA1 = 0.37 (0.19)	AR1 = 0.77 (0.12) Mean = 5.18 (0.18)	AR1 = 0.55 (0.17)	MA1 = 0.89 (0.17)	AR1 = 0.45 (0.20)	AR1 = 1.81(0.06) AR2 = −0.93(0.06) Mean = 61.62(0.82)	AR1 = 0.79 (0.11)	AR1 = 0.48 (0.18) Drift = 0.67 (0.22)

ARIMA(p,d,q): Autoregressive integrated moving average model and (p,d,q) are the autoregressive order, the degree of differencing and the moving average order

SE: standard error

AR: Autoregressive parameter

MA: Moving average parameter

8 and

**Table 9:** Estimates with their standard errors of the ARIMA models for the years 1995 to 2017 for South Africa

	**Health spending per capita ($)**	**Health spending per GDP (%)**	**Source of health spending**			**Share of health spending**		
			Government spending per capita ($)	Prepaid private spending per capita ($)	Out-of-pocket spending per capita ($)	Government spending as share of total (%)	Prepaid private spending as share of total (%)	Out-of-pocket spending as share of total (%)
**Model**	ARIMA(0,2,1)	ARIMA(0,2,1)	ARIMA(1,1,0)	ARIMA(1,1,0)	ARIMA(0,2,0)	ARIMA(2,1,0)	ARIMA(2,1,)	ARIMA(0,2,0)
**Estimates (SE)**	MA1 = −0.47 (0.19)	MA1 = −0.67 (0.18)	AR1 = 0.87 (0.09)	AR1 = 0.49 (0.18)		AR1 = 1.33 (0.18) AR2 = −0.50 (0.18)	AR1 = 1.32 (0.18) AR2 = 0.52 (0.17	

ARIMA(p,d,q): Autoregressive integrated moving average model and (p,d,q) are the autoregressive order, the degree of differencing and the moving average order

SE: standard error

AR: Autoregressive parameter

MA: Moving average parameter

9.
