# Correction to: Future health spending forecast in leading emerging BRICS markets in 2030: health policy implications

**DOI:** 10.1186/s12961-022-00836-z

**Published:** 2022-03-17

**Authors:** Mihajlo Jakovljevic, Demetrios Lamnisos, Ronny Westerman, Vijay Kumar Chattu, Arcadio Cerda

**Affiliations:** 1 Institute of Advanced Manufacturing Technologies, Peter the Great St. Petersburg Polytechnic University, St Petersburg, Russia; 2 Institute of Comparative Economic Studies, Hosei University, Tokyo, Japan; 3 Department of Global Health Economics and Policy, Faculty of Medical Sciences, University of Kragujevac, Kragujevac, Serbia; 4 Department of Health Sciences, European University Cyprus, Nicosia, Cyprus; 5 Federal Institute for Population Research, Wiesbaden, Germany; 6 Department of Medicine, Faculty of Medicine, University of Toronto, Toronto, ON M5G 2C4 Canada; 7 Center for Transdisciplinary Research, Saveetha Institute of Medical and Technical Sciences, Saveetha University, Chennai, India; 8 Department of Community Medicine, Faculty of Medicine, Datta Meghe Institute of Medical Sciences, Wardha, India; 9 Faculty of Economics and Business, University of Talca, Talca, Chile

## Correction to: Health Research Policy and Systems (2022) 20:23 10.1186/s12961-022-00822-5

During the publication process an error was introduced in the affiliations of two authors.

Affiliations 6 and 7 were attributed to Mihajlo Jakovljevic, but these affiliations are for Vijay Kumar Chattu. The publisher apologizes to the readers and authors for the inconvenience caused. The original article [1] has been updated.
